# Ensuring the Safety and Security of Frozen Lung Cancer Tissue Collections through the Encapsulation of Dried DNA

**DOI:** 10.3390/cancers10060195

**Published:** 2018-06-11

**Authors:** Kevin Washetine, Mehdi Kara-Borni, Simon Heeke, Christelle Bonnetaud, Jean-Marc Félix, Lydia Ribeyre, Coraline Bence, Marius Ilié, Olivier Bordone, Marine Pedro, Priscilla Maitre, Virginie Tanga, Emmanuelle Gormally, Pascal Mossuz, Philippe Lorimier, Charles Hugo Marquette, Jérôme Mouroux, Charlotte Cohen, Sandra Lassalle, Elodie Long-Mira, Bruno Clément, Georges Dagher, Véronique Hofman, Paul Hofman

**Affiliations:** 1 Hospital-Integrated Biobank (BB-0033-00025), Université Côte d’Azur, CHU de Nice, 06001 Nice CEDEX 1, France; washetine.k@chu-nice.fr (K.W.); mehdi.kara-borni@etu.unice.fr (M.K.-B.); bonnetaud.c@chu-nice.fr (C.B.); felix.jm@chu-nice.fr (J.-M.F.); ribeyre.l@chu-nice.fr (L.R.); ilie.m@chu-nice.fr (M.I.); bordone.o@chu-nice.fr (O.B.); pedro.m@chu-nice.fr (M.P.); Priscilla.maitre@gmail.com (P.M.); tanga.v@chu-nice.fr (V.T.); lassalle.s@chu-nice.fr (S.L.); long-mira.e@chu-nice.fr (E.L.-M.); hofman.v@chu-nice.fr (V.H.); 2 Team 4, Institute of Research on Cancer and Aging of Nice (IRCAN), Inserm U1081, CNRS UMR7284, Université Côte d’Azur, CHU de Nice, 06107 Nice CEDEX 2, France; heeke.s@chu-nice.fr (S.H.); bence.c@chu-nice.fr (C.B.); marquette.c@chu-nice.fr (C.H.M.); 3 Laboratory of Clinical and Experimental Pathology, Université Côte d’Azur, CHU de Nice, University Hospital Federation OncoAge, 06001 Nice CEDEX 1, France; 4 FHU OncoAge, University of Nice Sophia Antipolis, 06001 Nice CEDEX 1, France; 5 Université de Lyon, UMRS 449; Laboratoire de Biologie générale, Université Catholique de Lyon; Reproduction et développement comparé, EPHE, 69002 Lyon, France; egormally@univ-catholyon.fr; 6 Biobank of Grenoble (BB-0033-00069), 38043 Grenoble, France; PMossuz@chu-grenoble.fr (P.M.); PLorimier@chu-grenoble.fr (P.L.); 7 Department of Pulmonary Medicine and Oncology, Université Côte d’Azur, CHU de Nice, University Hospital Federation OncoAge, 06001 Nice CEDEX 1, France; 8 Department of Thoracic Surgery, Université Côte d’Azur, CHU de Nice, University Hospital Federation OncoAge, 06001 Nice CEDEX 1, France; cohen.c@chu-nice.fr (C.C.); mouroux.j@chu-nice.fr (J.M.); 9 INSERM INRA, NuMeCan, Rennes University, CRB Santé, CHU Rennes, 35000 Rennes, France; bruno.clement@inserm.fr; 10 INSERM, 75654 Paris, France; georges.dagher@inserm.fr

**Keywords:** lung cancer, tumor tissues, biobank, research projects, sustainability, DNA, genomic, personalized medicine, international networks, security

## Abstract

Collected specimens for research purposes may or may not be made available depending on their scarcity and/or on the project needs. Their protection against degradation or in the event of an incident is pivotal. Duplication and storage on a different site is the best way to assure their sustainability. The conservation of samples at room temperature (RT) by duplication can facilitate their protection. We describe a security system for the collection of non-small cell lung cancers (NSCLC) stored in the biobank of the Nice Hospital Center, France, by duplication and conservation of lyophilized (dried), encapsulated DNA kept at RT. Therefore, three frozen tissue collections from non-smoking, early stage and sarcomatoid carcinoma NSCLC patients were selected for this study. DNA was extracted, lyophilized and encapsulated at RT under anoxic conditions using the DNAshell technology. In total, 1974 samples from 987 patients were encapsulated. Six and two capsules from each sample were stored in the biobanks of the Nice and Grenoble (France) Hospitals, respectively. In conclusion, DNA maintained at RT allows for the conservation, duplication and durability of collections of interest stored in biobanks. This is a low-cost and safe technology that requires a limited amount of space and has a low environmental impact.

## 1. Introduction

Lung cancer is the major cause of death by cancer in the world and is an important health care issue. Campaigns for prevention, screening and new therapeutic strategies have not significantly reduced the annual incidence of this cancer, for which an increase is now observed for women [1,2,3]. Aside from tobacco smoking, the epidemiology of lung cancer points increasingly to exogenous factors [1,4]. Genetic predisposition can also participate in the development of this cancer [5,6,7]. Research into this field of thoracic oncology is strongly increasing. The discovery and validation of diagnostic, prognostic and predictive biomarkers is a high priority of the work of academic scientists and of the pharmaceutical industry in this domain [8,9,10,11,12,13,14,15,16,17]. The identification of predictive biomarker is mandatory for the use of companion diagnostic tests for administration of targeted treatments. Thanks to improvements in technologies that optimize biological analyses, which are increasingly sensitive and specific, the understanding of thoracic oncology is progressing. However, they are becoming more and more complex, in particular for high throughput sequencing methods. Regardless of the method of use, it is indispensable to perform analyses on high quality clinically indexed biological samples [18].

The major issue for the coming years certainly concerns the integration of epidemiological, clinical and biological data for discovery of biomarkers, in particular for precision medicine [19,20]. The use of this data for analyses including «deep learning» require more and more storage space for maintenance of the information [21,22,23,24,25]. Technology continues to evolve over time and the information generated from biological samples, in particular the DNA and other by-products, increases exponentially with time. The storage and securing of this information will become more and more costly and their safeguard is not risk-free. This has led to speculate that it is more appropriate to keep the DNA as a «reservoir» of information, rather than the information derived from already available analyses.

The strategy of collection of biological samples of lung cancers must take into account novel approaches to biomedical research, and thus the needs of scientists. A large number of samples of tissues and liquids from patients with lung cancer are conserved in biobanks. Beyond the need for quality and for associated clinical data, the samples do not always fulfill the criteria for inclusion into certain research projects. It may be necessary to make available samples associated to pathology or a rare clinical context for discovery and then validation of biomarkers present in only certain patients. This is the case for example, for biological samples of lung cancers from non-smokers and/or from small-sized tumors that metastasize very rapidly, or corresponding to rare histological subtypes (such as sarcomatoid carcinomas). So, it is difficult to include and use these samples in research projects concerning these pathologies. It is for this reason that optimal securitization and safety of these samples is indispensable to avoid their loss consequent to degradation or destruction following an incident such as an electricity failure, flood, fire, natural disaster or human error [26,27,28,29,30,31]. A secure system includes the installment of an alarm system and electrical safeguard, controlled access and empty freezers or liquid nitrogen containers to which the collection can be transferred in the event of equipment failure. However, regardless of the type of security deployed on a single storage site, the loss of samples in the event of a major catastrophe or poor management of an emergency (flood, earth quake, general electrical failure or human malpractice) cannot be avoided [26,29,30,32,33]. Only duplication of the biological collection and storage at a distance from the primary biobank at a controlled site (called «mirror» sites) assures an optimal safeguard [34,35]. The duplicated collection must be stored at «secondary» sites, respecting the same obligations and norms of quality of the «primary» site of storage.

Despite the recommendations of the different international organizations (EOCDI, NCI) the set-up of duplicated collections by biobanks is not much valued [34]. This procedure is thus not or very rarely reported by the directors/heads of biobanks. There are a number of reasons for this, including: (i) the cost; (ii) the difficulty in set up; and (iii) the need for storage space at low temperature at one or several secondary sites that are located at a distance to the primary biobank and possess the same norms of quality.

This study describes the set-up of the secure duplication of several collections of lung cancers selected according to their rarity and/or potential interest for use in research projects. We describe the different steps leading to the storage of lyophilized (dried) and encapsulated DNA conserved at RT at different «mirror» sites. We present the advantages and constraints of this strategy following adoption by the biobank of the Nice University Hospital Center (BB-0033-00025, Nice, France).

## 2. Results

### 2.1. Patients and Selected Samples for DNA Extraction and Selection of DNA for the Biobank of the Nice University Hospital Center

The selected patients and their main characteristics are shown in Table 1, Table 2 and Table 3. Selection from the database (which has information from 3241 lung cancer patients) included 331 non-smoking patients with NSCLC, 2001 smoker patients with early stage (I–II) cancer and 155 patients with a sarcomatoid carcinoma. After selection according to the above defined criteria a total of 1974 cryotubes of extracted and quantified DNA were sent to the Imagene platform.

### 2.2. Encapsulation of DNA (Imagene, Evry)

**Figure 1:**
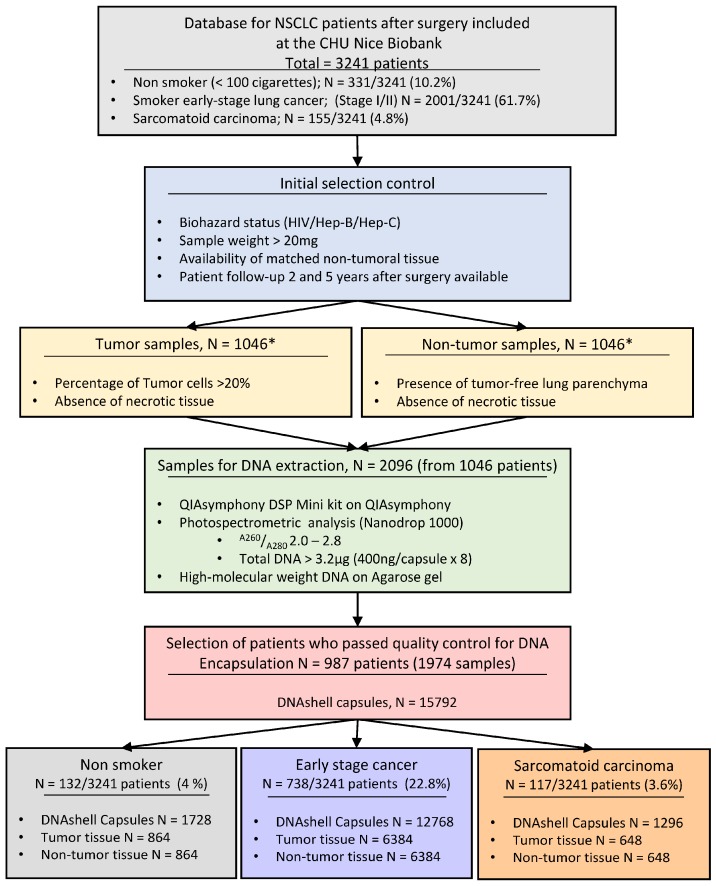
The different steps leading from the selection of patients and frozen tissue samples to DNA quality and quantity controls to DNA encapsulation. NSCLC = Non-Small Cell Lung Carcinoma. * The total number of selected cases (987) corresponds to 132 non-smoker patients plus 738 early stage carcinoma from smoker patients plus 117 sarcomatoid carcinoma histological subtypes.

All the DNA samples from the biobank of the Nice University Hospital School passed the quality control performed by the Imagene platform. Eight capsules of DNA (400 ng per capsule) were obtained based on the criteria defined above. A total of 15,792 contained each 400 ng of DNA capsules were sent to the biobank of the Nice University Hospital Center and placed in the storage cabinet. A selection of 3948 capsules (two capsules for each selected sample) was made and sent to the Grenoble University Hospital Center. The different steps are summarized in Figure 1.

**Figure 2:**
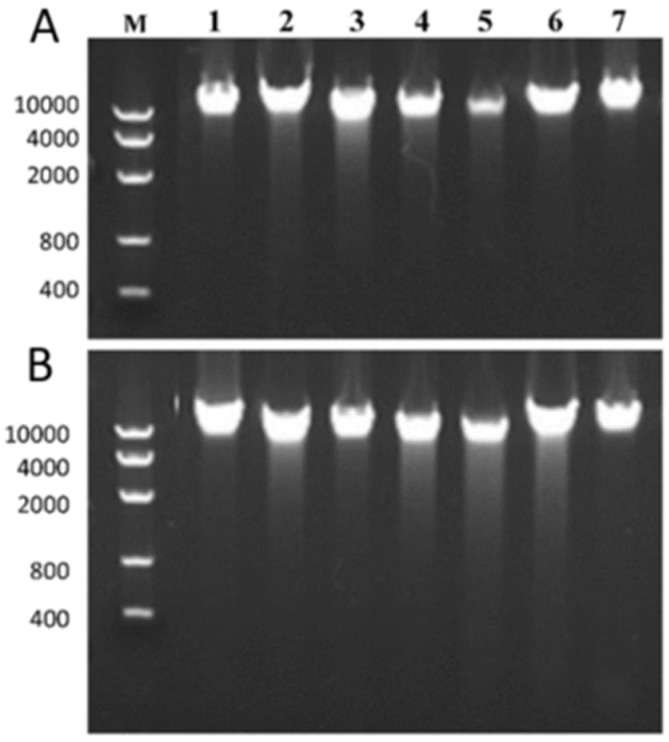
Comparative gel migration profiles from frozen (**A**) and corresponding dried (**B**) DNA which was extracted from 7 different tumor samples (1–7). M: Marker with the basepair length indicated next to the picture. DNA extracted from frozen tissue (**A**) as well as corresponding DNA extracted from encapsulated DNA (**B**) showed a strong band at high molecular weight for all 7 tumor samples indicating the presence of non-degraded, high quality DNA.

Finally, comparison of DNA quality in matched frozen and dried DNA samples showed similar results to those in Figure 2. Comparative gel migration from 7 matched dried and frozen DNA samples showed quite similar profiles with DNA of high molecular weights. Both dried and frozen DNA samples demonstrated similar A260/A280 ratio numbers (2.1 + 0.04 versus 2.1 + 0.06 for dried versus frozen DNA respectively) (not shown).

## 3. Discussion

Since its creation in 2004, the biobank of the Nice University Hospital Center (BB-0033-00025, Nice, France) has collected mainly lung cancers. The duplication of the total collection was not envisaged for strategic reasons because of the cost required. Initially the choice to duplicate the samples concerned three domains of interest. The first concerned lung cancers from non-smokers. Samples from these patients are often requested for research projects [36,37,38,39,40,41]. We anticipated that increasing interest in providing these samples to scientists would take place. The second domain concerned early stage lung cancers in smoker patients. Among these small-sized tumors, the patients who relapse two and five years after surgery and those who did not relapse at least five years after surgery were identified. At present one of the major issues in thoracic oncology is to define novel biomarkers that predict the evolution of lung cancers of small size [42,43,44,45,46,47]. In fact, in the future adjuvant treatments could probably be proposed to patients with tumors with a biological signature indicative of poor prognosis [48]. The third domain selected concerned a rare histological sub-type of NSCLC, sarcomatoid carcinomas [49]. The phenotype and genotype of these tumors is particularly notable and their prognosis is generally worse than that of most of the other types of lung adenocarcinomas [49,50,51].

**Figure 3:**
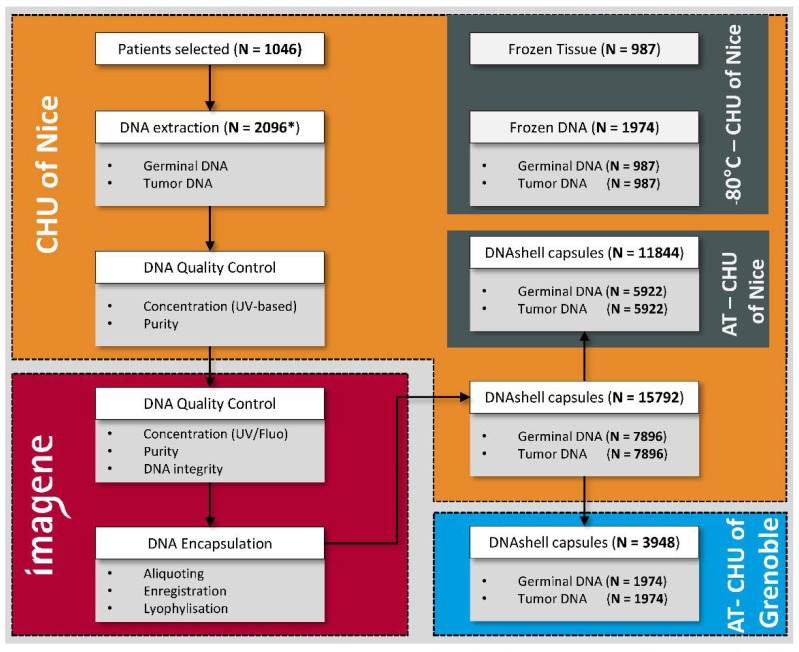
Workflow of the selected samples for duplication and storage in different sites. * For each patient, two samples have been selected: one tumor sample and one healthy tissue sample.

After selection of the frozen samples following the criteria mentioned above, the quality and quantity of the extracted DNA was evaluated before: (i) lyophilization, encapsulation and storage at RT and (ii) sampling in parallel into cryotubes for storage at −80 °C. The samples of DNA were thus conserved at three different geographical sites to guarantee maximum security in the event of an incident occurring at one of the sites. Two zones of storage were located at the Nice University Hospital Center (frozen DNA and DNA kept at RT) and the third at the Grenoble University Hospital Center, which is locate around 250 km from Nice (DNA kept at RT) (Figure 3). Frozen tissue samples from the same patients that were not used for DNA extraction were also stored at −80 °C in the Nice biobank. For each patient, tumor DNA and germ line DNA extracted from matched non-tumor lung tissue was obtained from the same patient and conserved. Beyond the possibility of looking for constitutional anomalies, this germ line DNA may allow the optimization of analyses of tumors by whole exome sequencing.

The duplication of a biological collection can concern frozen or fixed tissue samples or their by-products (nucleic acids and proteins). In fact, possession of: (i) one or several samples of adequate quality and quantity; (ii) freezers (−80 °C; −150 °C) or liquid nitrogen containers for the samples; (iii) an additional area of storage (mirror site) with the same security and quality norms as the primary biobank is essential when frozen tissues need to be duplicated and stored. Finally, duplication of frozen tissue samples can only be done at low temperatures, while duplication of nucleic acids can be done at low temperatures and/or at RT.

The collection of tissues or of frozen by-products is useful for some research projects in oncology but more and more requests for fixed tissue included in paraffin blocks and for nucleic acids, in particular DNA, are being made by research scientists. The mid- and long-term stability of the quality of the samples conserved at low temperature is often difficult to maintain and control, requiring substantial precautions [52,53,54]. It is for this reason that several procedures have been developed to maintain the quality and stability at RT of nucleic acids over a long period of time for subsequent use in research [55,56,57,58,59,60,61].

To identify novel biomarkers, more and more complex molecular analyses associated to clinically data are required. Research into genomic alterations of cancers or constitutional genetic polymorphisms is performing with nucleic acids [62]. An increasing number of molecular analyses using nucleic acids are required for personalized medicine and for development of novel therapeutic targets [62]. Collections of fixed tissue included in paraffin blocks can be used for both morphological analyses and molecular analyses after extraction of nucleic acids. However, the setup of collections must comply with regulatory constraints due to the extensive zone of storage and the security that requires continuous control of the temperature and hygrometry of these zones. In fact, as a consequence of variations in temperature and/or the degree of hygrometry or in the case of exposure to strong light, the tissue blocks can undergo a process of oxidation and degradation. Recent studies have also shown that degradation of tumor DNA in tissue blocks stored at RT can occur after several years of conservation [63,64,65]. Recent DNA sequencing approaches are very sensitive and so can lead to artifacts resulting from the long-term storage of tissues conserved in paraffin blocks [64]. For some biotechnologies using a broad panel of genes, it is also recommended that tissues stored for less than six months be used for analysis of DNA of patients (Foundation Medicine, Cambridge, MA, USA). Thus, for the future care of patients it appears necessary to store DNA in its «native» state. This is particularly true since we do not master the evolution of knowledge and techniques that may lead to the discovery of novel mechanisms of regulation and/or of molecular structure. Thus, it seems logical to be able to conserve over a very long period, in a secure and cost-free manner, DNA extracts by lyophilization and maintenance in capsules at RT.

The conservation of lyophilized, encapsulated and RT stored DNA holds several advantages compared to DNA extracted from frozen tissue, or kept at low temperature, or present in frozen tissues or in paraffin blocks (Table 4). Moreover, different technologies exist to maintain DNA at RT, but the DNAshell presents certain advantages in comparison with the other available technologies (Table 4) [56,58,66,67]. Encapsulated DNA is stable while DNA kept at low temperature can become modified depending on the conditions and period of storage [68,69,70,71,72]. Successive cycles of thawing and freezing of tissues do not allow optimal conservation of the integrity of the nucleic acid [54]. In the event of an electrical failure the samples stored at low temperature must be rapidly transferred to another storage location to avoid rapid and irreversible degradation. Once encapsulated the cost of conservation of the DNA is substantially lower than for storage at low temperature. The space required for encapsulated DNA is less than for storage of cryotubes at low temperature in freezers or in liquid nitrogen containers. The maintenance and servicing of the equipment, the electricity consumed, the security system (alarm) and the requirement for an additional zone for storage of samples in defective freezers and liquid nitrogen containers leads to costs that are higher than that for conservation of DNA at RT [73,74]. Storage at RT has almost no impact on the environment compared to that of storage at low temperature [75]. Finally, the transport of samples of encapsulated DNA at RT is less costly and the encapsulated DNA is not sensitive to variations in temperature, in comparison with more poorly controlled transport at low temperature [56,68,71].

When comparing duplication and conservation of samples at low temperature with duplicating and conservation at RT, a number of constraints exist (Table 4). Aside from the cost of extraction and quality control of the DNA (identical in the case of duplication of frozen samples), the cost of lyophilization and encapsulation of the DNA is, at present, higher than that for DNA duplicated and conserved in cryotubes. The cost also depends also probably on the number of samples encapsulated. However, if there is an increase in the demand for encapsulation the cost will most certainly decrease. Once the capsule is opened the lyophilized DNA is suspended in solution for use in projects and can no longer be kept at RT but can be frozen at low temperature, which may result in subsequent use of degraded DNA.

However, as some important information on the tissue is lost during the DNA extraction, additional information, such as the histological subtype, the percentage of tumor cell content, some results obtained by immunohistochemistry, the percentage of necrotic area and the immune cell infiltration and component in the stroma have to be stored in order to be able to make correct data interpretation, even after many years of storage.

The perpetuation of biobanks relies closely on a strategy evaluating the budgets and costs. The expenses concern to a large extent the costs of storage and securing of the biological samples. This budget line can be one of highest of the functioning of a biobank [74,76,77,78,79]. Encapsulation and conservation of DNA at RT can facilitate the setup of a « mirror » site for storage (economize energy, reduced space requirements, decrease in the need to replace equipment). However, regardless of the method of packaging and of storage of extracted DNA, all the pre-analytical steps must be perfectly managed, and the quality and quantity of DNA must be controlled before encapsulation [63,65].

## 4. Materials and Methods

The clinical-biological database of the biobank was querying for a period of 12 years (2004–2016) for NSCLC patients. The aim was to select frozen samples meeting the following criteria: (i) tumors from non-smoker patients (less than 100 cigarettes smoked); or (ii) early stage tumors from smoker patients, according to the WHO classification or (iii) sarcomatoid carcinomas as classified by the WHO [42,80]. The protocol was approved by the ethical committee of the University Côte d’Azur and the Nice Hospital Center and all patients provided written informed consent. The study complies with the World Medical Association Declaration of Helsinki regarding ethical conduct of research involving human subjects.

The following information was then collected for all the selected patients: (i) the serological status for HIV, hepatitis B and C viral infections; (ii) the smoking history (non-smoker, former smoker, present smoker); (iii) the availability of matched frozen non-tumor and tumor tissues; (iv) the weight of the sample before freezing; (v) information on the follow up of patients, two then five years after surgery (absence of relapse, loco-regional progression, metastasis or death).

The samples of tumors were then selected according to the following criteria: (i) weight > 20 mg; (ii) percentage of tumor cells > 20% (evaluated on tissue sections stained with hematoxylin eosin obtained from formalin fixed mirror tissue blocks) and (iii) absence of necrotic tissue. The non-tumor frozen tissues were then selected according to the following criteria: (i) weight > 20 mg; (ii) presence of lung parenchyma; (iii) absence of tumor cells and (iv) absence of necrosis (evaluated as described above).

The extraction of DNA was performed on a Qiasymphony (Qiagen, Hilden, Germany) instrument using the QIAsymphony DSP Mini Kit (Qiagen). Control of the quality and the purity (A260/A280) was done by fluorimetric and spectrometric analysis with a NanoDrop 1000 (ThermoFisher Scientific, Waltham, MA, USA). A diluted sample of 200 µL was obtained. Half of this volume was sent to the Imagene (Genopole Campus, Evry, France) platform and the other half was stored at −80 °C in the biobank of the Nice University Hospital Center. Only samples with a purity, evaluated with the A260/A280 ratio, of between 2.0 ± 0.04 and 2.8 ± 0.9 and showing a high molecular weight on an agarose gel were quantified, conserved and stored at 4 °C before transport to Imagene. The steps leading to the selection of the DNA are shown in Figure 1.

The extracted and quantified DNA was sent at 4 °C to the Imagene (Genopole Campus 1, Evry, France) platform. Additional control was performed on reception of the samples by fluorimetric and spectrometric analysis, by evaluation of the purity (A260/A280) and electrophoresis on an agarose gel. An arbitrary number of eight capsules per sample was chosen, each containing a minimum of 400 ng of DNA. Encapsulation was performed as described previously [55,56,68,81]. Briefly, aliquots of the samples were made using a specifically configured instrument to avoid pipetting errors and contamination. The DNA was put into stainless steel capsules and subjected to lyophilization [55,56,68,81]. The capsules were labeled with a 2D code with a laser to assure tracking. The capsules were welded with a laser in a dehydrated and anoxic atmosphere. The tightness was verified at the end of encapsulation. All the capsules containing DNA were then sent at RT to the biobank of the Nice University Hospital Center and stored at RT in a dedicated cabinet (IMAGENE SRS 268816, Imagene, Evry, France), located in a secure location at a distance to the primary biobank. Two capsules of each sample (corresponding to the non-tumor and tumor samples) were then sent at RT for storage in the biobank of the Grenoble University Hospital Center.

It was previously and extensively shown that dried and encapsulated DNA (either purified or within the sample source) is very stable and that this form of storage yields DNA compatible with downstream analyses [56,68,81]. We did a comparison of DNA quality in matched frozen and dried DNA samples obtained from the capsules stored in the biobank. These DNA samples took at random from 7 cases among all cases included in this work were extracted from the same tumor tissues. The samples were then analyzed on a 1% agarose gel using the E-Gel Precast Agarose Electrophoresis System (ThermoFisher Scientific, Waltham, MA, USA).

## 5. Conclusions

In conclusion, this study shows that the safeguarding of a collection of interest is optimal if duplication and storage can be done in an area that assures its security, ideally at a location distant from the primary site of storage. It is easier to duplicate DNA than frozen tissues or formalin fixed paraffin embedded tissue blocks. The initial cost of duplication of DNA by lyophilization and encapsulation for RT storage is higher than that for duplication of frozen DNA but in the mid-term this economic model is most certainly optimal and the security of storage and of transport of the samples is strongly increased. In addition, this technique of encapsulation and conservation at RT can also be used for storage of lyophilized RNA [81,82,83]. This nucleic acid is more fragile than DNA and the impact of the different pre-analytical steps including the variation in temperature are important. In total, investment in this technique of conservation of nucleic acids may be sound in the mid-term for both scientific reasons and for setup of a model economic biobank. Importantly, DNA can also be considered as a stable support of information. The storage and safeguard at RT is certainly cheaper and safer than storage of computer databases of derived information. Rather than investing in big data centers, dry-encapsulated DNA associated with computer chip could become the simplest and most effective information storage systems in medical genomics and public health.

## Figures and Tables

**Table 1:** Main epidemiological parameters associated with the cohort of non-smoker lung cancer patients (total of 132 patients).

Variables	*n* (%)
**Age (Years)**	
Mean (range)	62 (36–82)
**Sex**	
Male	72 (55%)
Female	60 (45%)
**Tumor Size (cm)**	
Mean (range)	3.5 (1–9)
**pTNM Stage**	
I	42 (32%)
II	61 (46%)
III	30 (23%)
IV	9 (7%)
**Histological Subtype**	
Adenocarcinoma	117 (87%)
Squamous cell carcinoma	8 (6%)
Other	7 (7%)
**Mutational Status**	
*EGFR* mutation	26 (20%)
*ALK* rearrangement	15 (11%)
*ROS1* rearrangement	5 (4%)
*BRAF* mutation	5 (4%)

**Table 2:** Main epidemiological parameters associated with the cohort of early-stage NSCLC patients (total of 738 former or current patients).

Variables	*n* (%)
**Age (Years)**	
Mean (range)	67 (38–81)
**Sex**	
Male	525 (71%)
Female	213 (29%)
**Smoking Status**	
Current	631 (85%)
Former	107 (15%)
**Tumor Size (cm)**	
Mean (range)	3.5 (1-5.5)
**pTNM Stage**	
Ia	37 (5%)
Ib	146 (20%)
Ic	160 (21%)
IIa	203 (28%)
IIb	192 (26%)
**Histological Subtype**	
Adenocarcinoma	461 (62%)
Squamous cell carcinoma	196 (27%)
Other	81 (11%)
**Follow-Up 2 Years after Surgery**	
Metastasis	28 (3%)
Death related to lung cancer	27 (3%)
**Follow-Up 5 Years after Surgery**	
Metastasis	99 (12%)
Death related to lung cancer	68 (8%)

**Table 3:** Main epidemiological parameters associated with the cohort of lung sarcomatoid carcinoma patients (total of 117 patients).

Variables	*n* (%)
**Age (Years)**	
Mean (range)	68 (41–79)
Sex	
Male	80 (68%)
Female	37 (22%)
**Tobacco Status**	
Current/former	95 (81%)
Former	22 (19%)
**Tumor Size (cm)**	
Mean (range)	4.2 (2.3–9)
pTNM stage	
I	12 (%)
II	55 (%)
III	35 (%)
IV	15 (%)
**Histological Subatype**	37 (32%)
Pleomorphic carcinoma	35 (30%)
Spindle cell carcinoma	26 (22%)
Giant cell carcinoma	8 (7%)
Carcinosarcoma	6 (5%)
Pulmonary blastoma	5 (4%)

**Table 4:** Advantages and disadvantages of DNA storage at room temperature according to different technologies versus frozen procedure.

DNA Storage at Room Temperature			Frozen DNA Storage
DNA Shells	DNA Stabilization Matrices	DNA Cards	
Dried DNA stored encapsulated in minicapsules.	Dried DNA stored in tubes.	Dried DNA stored in cards.	DNA stored in water or low concentrated TE buffer at −80 °C.
***Chemical Stability***			
Solid state reduces chemical reactivity and limits hydrolysis and oxidation. Encapsulation completely protects DNA from moisture and oxygen. Long DNA fragments and a broad range of DNA amount can be stored.	Solid state reduces chemical reactivity and limits hydrolysis and oxidation. Trace amounts of DNA can be stored **BUT** DNA is exposed to atmospheric influences. Moisture and temperature have to be controlled.	Solid state reduces chemical reactivity and limits hydrolysis and oxidation. **BUT** DNA is exposed to atmospheric influences. Moisture and temperature have to be controlled.	Reduced chemical reactivity due to reduced storage temperature **BUT** Storage in aqueous solution potentially allows hydrolysis and oxidation.
***Storage***			
Energy saving and automation friendly Standalone storage system.	Energy saving and automation friendly. Standalone storage system.	Energy saving and automation friendly. Standalone storage system.	Storage devices are usually already available **BUT** Energy consuming and high-maintenance devices are needed. Backup systems need to be provided and maintained.
***Sample Shipment***			
Samples can easily be shipped.	Samples can easily be shipped but moisture should be controlled if long transportation time is expected.	Samples can easily be shipped but moisture should be controlled if long transportation time is expected.	Shipment is complicated and risky as the low temperature has to be maintained during shipment.
***Handling***			
Easy and quantitative recovery possible **BUT** Dehydration and encapsulation have to be performed by external service providers and initial costs are high.	Easy and quantitative recovery possible. DNA drying can be easily performed at the customer lab without additional devices needed **BUT** Special tubes are needed and storage location has to be controlled for temperature and moisture.	DNA preservation can easily be performed at the customer lab without additional devices needed. **BUT** Quantitative recovery is not possible and storage location has to be controlled for temperature and moisture.	Normal and cheap cryotubes can be used for storage and freezing of DNA is quite easy **BUT** The protocol is not adapted to trace amounts of DNA and sample concentrates over time due to sublimation of storage buffer.
